# Neurostructural Differences in Adolescents With Treatment-Resistant Depression and Treatment Effects of Transcranial Magnetic Stimulation

**DOI:** 10.1093/ijnp/pyac007

**Published:** 2022-01-28

**Authors:** Bhedita J Seewoo, Jennifer Rodger, Mark A Demitrack, Karen L Heart, John D Port, Jeffrey R Strawn, Paul E Croarkin

**Affiliations:** Experimental and Regenerative Neurosciences, School of Biological Sciences, The University of Western Australia, WA, Australia; Brain Plasticity Group, Perron Institute for Neurological and Translational Science, WA, Australia; Centre for Microscopy, Characterisation and Analysis, Research Infrastructure Centre, The University of Western Australia, Perth, WA, Australia; Experimental and Regenerative Neurosciences, School of Biological Sciences, The University of Western Australia, WA, Australia; Brain Plasticity Group, Perron Institute for Neurological and Translational Science, WA, Australia; Mayo Clinic, Rochester, Minnesota, USA; Trevena, Inc. Chesterbrook, Pennsylvania, USA; Advicenne, Inc., Greater Philadelphia, PA, USA; Department of Radiology Chesterbrook, Pennsylvania, USA; Department of Psychiatry and Psychology Chesterbrook, Pennsylvania, USA; Department of Psychiatry and Behavioral Neuroscience, University of Cincinnati, Cincinnati, Ohio, USA; Department of Psychiatry and Psychology Chesterbrook, Pennsylvania, USA

**Keywords:** Adolescent, amygdala, magnetic resonance imaging, transcranial magnetic stimulation, treatment resistant depression

## Abstract

**Background:**

Despite its morbidity and mortality, the neurobiology of treatment-resistant depression (TRD) in adolescents and the impact of treatment on this neurobiology is poorly understood.

**Methods:**

Using automatic segmentation in FreeSurfer, we examined brain magnetic resonance imaging baseline volumetric differences among healthy adolescents (n = 30), adolescents with major depressive disorder (MDD) (n = 19), and adolescents with TRD (n = 34) based on objective antidepressant treatment rating criteria. A pooled subsample of adolescents with TRD were treated with 6 weeks of active (n = 18) or sham (n = 7) 10-Hz transcranial magnetic stimulation (TMS) applied to the left dorsolateral prefrontal cortex. Ten of the adolescents treated with active TMS were part of an open-label trial. The other adolescents treated with active (n = 8) or sham (n = 7) were participants from a randomized controlled trial.

**Results:**

Adolescents with TRD and adolescents with MDD had decreased total amygdala (TRD and MDD: −5%, *P* = .032) and caudal anterior cingulate cortex volumes (TRD: −3%, *P* = .030; MDD: −.03%, *P* = .041) compared with healthy adolescents. Six weeks of active TMS increased total amygdala volumes (+4%, *P* < .001) and the volume of the stimulated left dorsolateral prefrontal cortex (+.4%, *P* = .026) in adolescents with TRD.

**Conclusions:**

Amygdala volumes were reduced in this sample of adolescents with MDD and TRD. TMS may normalize this volumetric finding, raising the possibility that TMS has neurostructural frontolimbic effects in adolescents with TRD. TMS also appears to have positive effects proximal to the site of stimulation.

**Significance Statement**

To our knowledge, this is the first neurostructural examination of treatment-resistant depression in adolescents (TRD). Adolescents with TRD and adolescents with a current major depressive disorder (MDD) had decreased amygdala volumes compared with healthy adolescents. Further, left prefrontal, high frequency transcranial magnetic stimulation (TMS)—but not sham TMS—corrected these decreased amygdala volumes in adolescents with TRD. Treatment with active, left prefrontal, high-frequency TMS also increased dorsolateral and dorsomedial prefrontal cortex volumes in this sample of adolescents with TRD.

## Introduction

More than one-third of adolescents with major depressive disorder (MDD) fail to respond to initial treatment with selective serotonin reuptake inhibitors (SSRIs) or cognitive behavioral therapy and thus meet the definition of treatment-resistant depression (TRD) (Emslie et al., 2002; Weisz et al., 2006; Bridge et al., 2007). Although TRD in adolescents is common clinically, it has been poorly characterized and potential treatments are understudied. There are ongoing debates regarding the definition of TRD in adolescents. Often, the definition diverges from how TRD is characterized in adults, and this presents additional challenges in designing and interpreting studies in adolescents (Dwyer et al., 2020; Strawn and Croarkin, 2020). For example, few clinical trials have focused on adolescents with TRD, and the neurobiological characteristics of TRD in adolescents have not been adequately characterized (Brent et al., 2008; Strawn et al., 2020). Transcranial magnetic stimulation (TMS) is an emerging treatment for adolescents with TRD (Croarkin et al., 2021). The mechanistic aspects of TMS in adolescents with TRD are also understudied (Croarkin et al., 2016a; Croarkin and Rotenberg, 2016).

The most recent studies of adolescents with MDD that does not responds to an initial trial of an SSRI suggest that an additional SSRI trial may have positive clinical effects (Suresh et al., 2020). However, there is a well-documented risk of transient increased suicidality with SSRIs in adolescents and young adults. Treatment with TMS is a potential alternative in the context of the concerns related to SSRIs and increased suicidality (Miller and Campo 2021). Ketamine has also been explored as an emerging intervention for TRD in adolescents, although only short-term data exist (Dwyer et al., 2021). Electroconvulsive therapy (Pierson et al., 2021), augmentation with mixed dopamine serotonin receptor antagonists, and augmentations with stimulant medications are additional options (Whitlock et al., 2020), but data from controlled trials are lacking. Treatment with TMS may have a favorable side effect burden and lower risks for adolescents compared with commonly used augmentation agents (Bobo et al., 2013). Examining neuromodulation-based treatments such as TMS has been challenging because work with biomarkers in adolescents with TRD is limited (Croarkin et al., 2021). Preliminary work suggests that impaired gamma-aminobutyric acid receptor B-mediated inhibition as assessed with neurophysiological measures is associated with TRD in adolescents (Croarkin et al., 2014; Lewis et al., 2018). Another study demonstrated decreased right superior temporal gyrus volumes in adolescents with TRD who had previously attempted suicide (McLellan et al., 2018). These prior studies are important but do not account for treatment effects or duration of illness and are based on historical traits. Prior clinical and preclinical literature focused on adults with MDD and TRD suggests that the prefrontal cortex and limbic structures such as the amygdala have a key role in the pathophysiology of depression (Pizzagalli and Roberts, 2022).

To address these knowledge gaps, we examined structural MRI studies from adolescent patients with TRD with 3 broad goals. First, using automatic segmentation and parcellation in FreeSurfer, baseline volumetric measures were examined in healthy adolescents, adolescents with MDD, and adolescents with TRD with the goal of further explication of the adolescent TRD phenotype. A subsample of adolescents with TRD were treated with 6 weeks of active or sham left prefrontal, high-frequency TMS and also had pre– and post–brain MRI measures. Finally, pre– and post–brain MRI neurostructural treatment effects of TMS were evaluated. We hypothesized that adolescents with TRD would demonstrate decreased frontal and limbic volumes compared with both adolescents with MDD and healthy adolescents. It was further hypothesized that TMS treatment for adolescents with TRD would attenuate structural deficits in frontal and limbic volumes.

## MATERIALS AND METHODS

### Participants

Prior study samples (NCT01502033, NCT02307617, NCT02586688, and NCT02818751) from 2 academic adolescent psychopharmacology research programs were pooled to obtain the study group. The pooled studies had similar protocols and recruitment processes. Depressed participants were recruited from within the clinics and via referrals from other care providers. Depressed and healthy participants were recruited with radio advertisements, invitation letters sent to parents of potentially eligible participants, print advertisements, a trial listing with ClinicalTrials.gov, a trial listing on university and clinical study site website, and social media (Wall et al., 2016; Croarkin et al., 2016, 2021; Lewis et al., 2016; Lu et al. 2021).

The study group consisted of 30 healthy adolescents (age range 13–21 years), 19 adolescents with MDD (age range 11–19 years), and 34 adolescents with TRD (age range 12–20 years), who were recruited based on objective antidepressant treatment resistance rating criteria. The 12–21 age range was studied because this line of research is focused on adapting brain stimulation devices for adolescents, and this is the age range defined as adolescent in accordance with US FDA guidance (US Food and Drug Administration, 2014). All participants with TRD had at least 1 prior failed trial of antidepressant medications in the current depressive episode on the basis of Antidepressant Treatment History Form (Sackeim, 2001) standards. If there were insufficient numbers of trials in the current episode, then the participant must also have failed ≥1 and ≤4 trials in a previous episode. Participants who have been unable to complete an antidepressant trial of adequate dose and duration due to intolerance to antidepressant therapy may be included if they have demonstrated intolerance to ≥4 antidepressant medications within 1 discrete illness episode (current or a previous) (Wall et al., 2016; Croarkin et al., 2016, 2021).

A subsample of adolescents with TRD were treated with active (n = 18, age range 12–19 years) or sham (n = 7, age range 16–19 years) left prefrontal, high-frequency TMS as monotherapy (Strawn et al., 2020; Croarkin et al., 2021). Adolescents were evaluated and monitored by child and adolescent psychiatrists for the duration of the study. Demographics, inclusion criteria, and exclusion criteria have been reported elsewhere (Croarkin et al., 2016b, 2021). Local institutional review board approval was obtained prior to any research-related activities. Participants 12−17 years of age provided informed assent and their parents provided informed consent. Participants 18−21 years of age provide informed consent.

### Study Overview and Clinical Measures

All participants had a clinical interview with a board-certified child and adolescent psychiatrist (J.R.S. or P.E.C.). The diagnosis of depression was based on Diagnostic and Statistical Manual of Mental Disorders, Fifth Edition criteria (American Psychiatric Association and American Psychiatric Association DSM-5 Task Force, 2013) and an interview with either the Schedule for Affective Disorders and Schizophrenia for School-Age Children-Present and Lifetime version (Kaufman et al., 1997), the Mini International Neuropsychiatric Interview for Children and Adolescents (for participants 12−17 years of age) (Sheehan et al., 2010) or the Mini International Neuropsychiatric Interview (for participants 18−21 years of age) (Sheehan et al., 1998). Depressive symptom severity was assessed with the Children’s Depression Rating Scale, Revised (CDRS-R) (Poznanski et al., 1984), the Quick Inventory of Depressive Symptomatology Adolescent Self Report (Bernstein et al., 2010), and 24-item Hamilton Depression Rating Scale (Hamilton, 1960). The depressed participants had depressive symptom severity with a raw score of ≥40 on the CDRS-R, and this was an inclusion criterion in 2 studies. In the randomized controlled trial of TMS, the inclusion criterion was a 24-item Hamilton Depression Rating Scale 1 score of ≥2 with a total score of ≥20. Participants in the randomized controlled trial were also assessed with the CDRS-R and had a raw score of ≥40.

For participants with MDD, inclusion criteria had variable age ranges with 12−21 years. Participants in the randomized controlled trial of TMS were not taking antidepressant or psychotropic medications, whereas in the other studies this was allowed. Exclusion criteria across studies for contraindications to TMS were consistent and informed by international standards (Rossi et al., 2021). Any history of epilepsy, cardiac pacemakers, implanted medication pumps, and intracardiac lines were exclusionary. Any implanted electronic device, metal in the head, or unstable medical conditions were exclusionary. Significant acute risk for suicide (based on the principal investigator’s assessment) was exclusionary. Pregnancy or the inability to use an accepted method of birth control for females who were sexually active were additional exclusion criteria. Any comorbid psychotic disorder, bipolar disorder, active substance use, active eating disorders, obsessive compulsive disorder, or posttraumatic stress disorder were exclusionary.

### Magnetic Resonance Imaging (MRI)

Healthy baseline MRI scans from Lu et al. (2021) were acquired on an Achieva Philips MRI scanner with a 32-channel phased-array head coil using a 3-dimensional T1-weighted Turbo field echo sequence with repetition time (TR) = 6.8 ms, echo time (TE) = 2.9 ms, number of sagittal slices = 160, resolution = 1 mm, slice thickness = 1 mm, flip angle = 9°, and matrix = 256 × 256. Additional healthy baseline MRI scans and baseline scans from adolescents with MDD and TRD were acquired on a GE Discovery 750 MRI scanner with an 8-channel head coil using a 3-dimensional T1-weighted FAST Spoiled Gradient-Recalled sequence with TR = 7.4 ms, TE = 3.0 ms, number of sagittal slices = 124, resolution = 1.02 mm × 1.02 mm, slice thickness = 1.2 mm, FA = 8°, and matrix = 256 × 256 (Lewis et al., 2020). Baseline and post-TMS/post-active and sham scans from adolescents with TRD were also acquired on a GE Discovery 750 MRI scanner with an 8-channel head coil using a 3-dimensional T1-weighted FAST Spoiled Gradient-Recalled sequence with TR = 12.6 ms, TE = 5.6 ms, number of axial slices = 116, resolution = .49 mm × .49 mm, slice thickness = 1.5 mm, FA = 15°, and matrix = 512 × 512 (Croarkin et al., 2016b).

### Transcranial Magnetic Stimulation

The abductor pollicis brevis site on the motor cortex was identified with standard procedures, as described elsewhere (O’Reardon et al., 2007; George et al., 2010). The resting motor threshold (minimum power to produce a stimulation response over the motor cortex abductor pollicis brevis muscle area 50% of the time) was determined at baseline with a parameter estimation algorithm as described previously (O’Reardon et al., 2007; Croarkin et al., 2021). For 10 adolescents in an open-label trial, the L-DLPFC treatment site was identified with MRI under the supervision of a neuroradiologist (Wall et al., 2016, Croarkin et al., 2016). The L-DLPFC treatment site was identified with the 5-cm rule in 15 adolescents in the randomized controlled trial (Croarkin et al., 2021) to harmonize the methodology with a prior landmark study of adults (O’Reardon et al., 2007). Treatment sessions were delivered with a Neurostar Therapy System (Neuronetics, Inc., Malvern, PA , USA). Stimulation was applied to the L-DLPFC at 120% motor threshold and 10-Hz frequency. Stimulus trains were 4 seconds and inter-train intervals were 26 seconds, with 75 trains delivered over 37.5 minutes to provide a total of 3000 pulses every session. The sham coil was identical in its appearance to the active coil, operated with an acoustically matched profile that rendered the auditory experience of the treatments virtually indistinguishable to participants or researchers, and created a mild percussive sensation to further mimic the active condition. The sham coil did not provide electrical stimulation. Patients were offered the opportunity to complete up to 36 active treatment sessions over 6–9 weeks.

### Statistical Analysis

All statistical analyses were performed in RStudio v4.0.2. (R Studio Team, 2018). Group differences in the basic demographics were examined with a 1-way ANOVA for continuous variables (age and total CDRS-R scores), and Fisher’s Exact Tests were used for categorical variables (sex).

**Figure 1.**
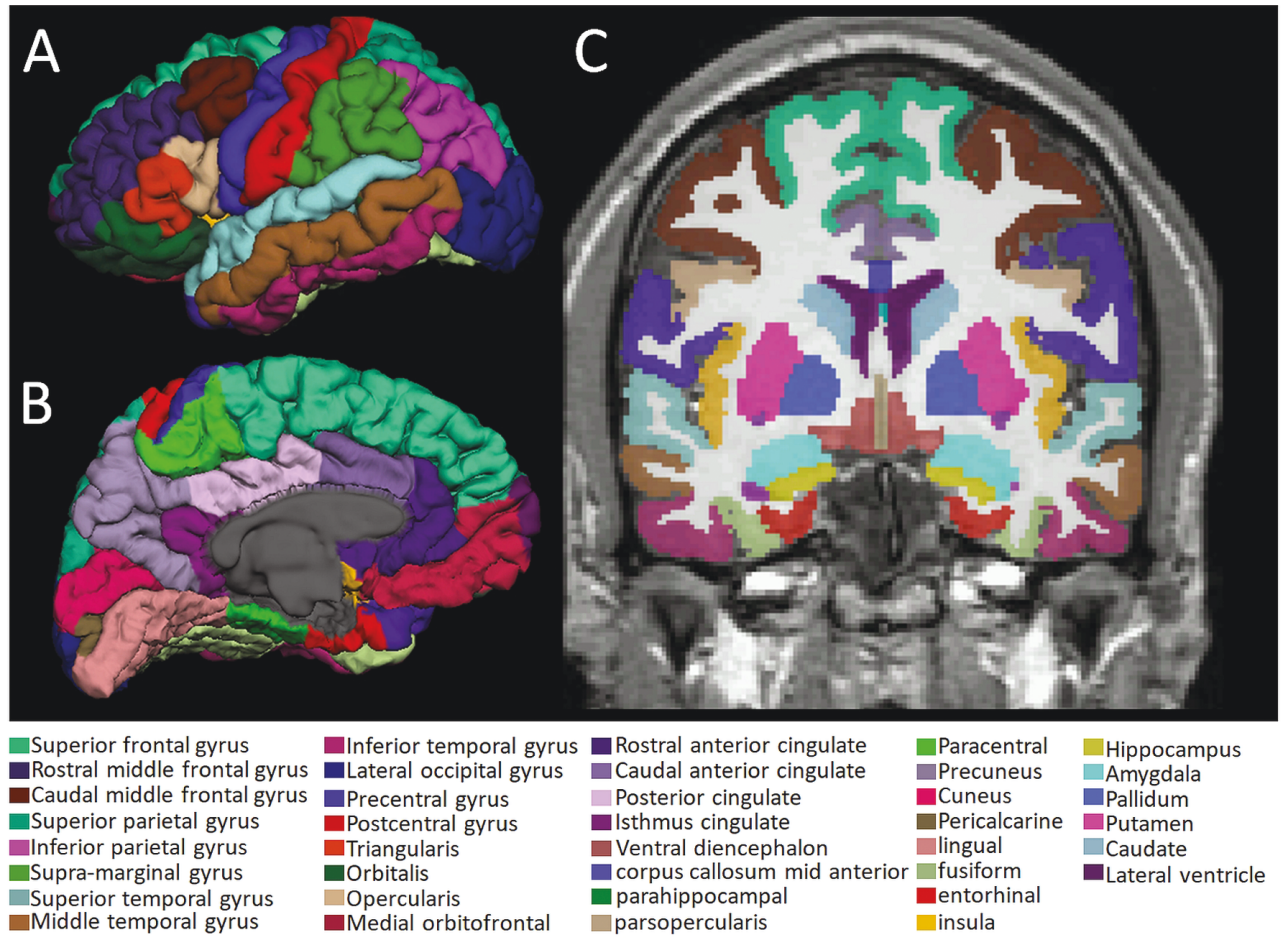
Visualization of the cortical and subcortical segmentation of T1-weighted anatomical data from a representative patient. The figure shows cortical parcellations (external surface) of the three-dimensional brain-extracted data (A and B) and segmentation of the cortical and subcortical structures (internal surface) overlaid on a coronal slice of the raw data (C). Each structure is labeled with a unique color distinction.

Image reconstruction and automated segmentation were carried out in FreeSurfer package 7.1.0 (http://surfer.nmr.mgh.harvard.edu) to eliminate intra- and inter-rater bias of manual tracing and maximize reproducibility. Quality control was performed by checking for outliers and using visual inspection as described in a recent protocol (http://enigma.ini.usc.edu/protocols/imaging-protocols/). Automated segmentation of whole amygdala into subnuclei was also carried out in FreeSurfer (Saygin et al., 2017). The following subfields were of interest: left/right/whole lateral nucleus, basal nucleus, central nucleus and medial nucleus. Estimated intracranial volume (ICV) and cortical and subcortical volumes were automatically derived from FreeSurfer. Cortical regions of interest included the DLPFC (FreeSurfer labels: superior frontal, rostral middle frontal, and caudal middle frontal gyri), the ventrolateral prefrontal cortex (FreeSurfer labels: pars opercularis, pars triangularis, and pars orbitalis), dorsomedial prefrontal cortex (FreeSurfer labels: superior frontal), caudal anterior cingulate cortex, and rostral anterior cingulate cortex. A visualization of the cortical and subcortical segmentations is shown in Figure 1.

To determine group differences, the raw volumes of all brain regions were normalized to individual ICV and reported as a percentage of ICV (Lehéricy et al., 1994). Type III ANCOVA (“car” package) was used to test for any effect of depression (Healthy vs MDD+TRD) on brain volumes with age, sex, and total CDRS-R scores as covariates. Post hoc comparisons were then carried out to analyze differences between healthy and MDD and TRD groups with age, sex, and total CDRS-R scores as covariates using the “emmeans” package. The false-discovery rate method was applied for multiple comparison correction, and *P* < .05 was considered significant. If significant, the comparison was repeated for all subfields.

To determine the effect of active and sham stimulation, paired comparisons were performed, and therefore, raw volumes were used in the analyses. The “lmer” and “emmeans” functions were used to analyze within-subject differences in brain volumes between timepoints with number of TMS sessions, age, sex, and total CDRS-R scores as covariates. *P* < .05 was considered significant. If significant for whole amygdala volume, the comparison was repeated for all subfields. For brain regions showing significant changes in volumes, the “emmeans” function was used to compare percentage change in volumes between active and sham groups with number of TMS sessions, age, sex, and total CDRS-R scores as covariates.

To determine whether volumetric changes following active stimulation were associated with clinical changes, Pearson or Spearman’s rank correlation (depending on normality of data) was carried out between change in volumes and change in depression scores of adolescents with TRD. Additionally, Spearman’s rank correlation was performed between the number of active TMS sessions patients with TRD received and the change in their depression scores.

## RESULTS

### Participant Characteristics

The demographics of the study cohort are shown in Table 1. The 3 groups (healthy, MDD, and TRD) did not differ in age (ANOVA, *F*_[2,80]_ = 2.924, *P* = .060) or sex (Fisher’s exact test, *P* = .595) distributions. A pooled subsample of adolescents with TRD were treated with 6 weeks of active (n = 18) or sham (n = 7) 10-Hz TMS applied to the L-DLPFC. Ten of the adolescents treated with active TMS were part of an open-label trial. The other adolescents treated with active (n = 8) or sham (n = 7) were participants from a randomized controlled trial. The mean number of TMS sessions completed was 32.1 ± 3.5 in the sham group (range 27−36), and 29.3 ± 13.3 (range 1–66) in the active group; 14 patients completed 29−36 sessions, and 1 each completed 66, 17, 5, and 1 sessions. The 2 subgroups did not differ in age (ANOVA, *F*_[1,23]_ = 2.282, *P* = .144), sex (Fisher’s exact test, *P* = 1), or total CDRS-R at baseline (ANOVA, *F*_[1,23]_ = 3.396, *P* = .078).

**Table 1.** Patient Characteristics

Characteristic	Healthy	MDD	TRD	Active TMS	Sham TMS
n	30	19	34	18	7
Female, n (%)	21 (70%)	11 (57.9%)	20 (58.8%)	12 (66.7%)	5 (71.4%)
Age, mean ± SD	15.6 ± 2.3	15.2 ± 1.8	16.4 ± 1.8	16.3 ± 2.0	17.5 ± 1.2
Minors (<18 y), n (%)	24 (80%)	18 (95%)	25 (74%)	14 (78%)	5 (71%)
Baseline CDRS-R, mean ± SD	19.1 ± 2.8	54.1 ± 8.2	59.6 ± 11.4	50.2 ± 15.1	61.4 ± 8.4
Episodes, n (%)					
Single	N/A	10 (52.6%)	8 (23.5%)	3 (16.7%)	1 (14.3%)
Recurrent	NA	9 (47.4%)	26 (76.5%)	15 (83.3%)	6 (85.7%)
Most recent episode duration (mo), mean ± SD	N/A	7.5 ± 8.5	15 ± 16.3	18.6 ± 18.5	8.6 ± 3.6
Past psychiatric hospitalizations, n (%)					
Yes	NA	5 (26.3%)	8 (23.5%)	5 (27.8%)	2 (28.6%)
No	NA	14 (73.7%)	26 (76.5%)	13 (72.2 %)	5 (71.4 %)
Lifetime suicide attempts, n (%)					
Yes	NA	5 (26.3%)	12 (35.3%)	5 (27.8%)	3 (42.9%)
No	NA	14 (73.7%)	22 (64.7%)	13 (72.2%)	4 (57.1%)
Prior medication trials based on ATHF scores, mean ± SD	NA	.4 ± .9	1.9 ± 1.8	2.7 ± 2.2	1 ± 0
Currently taking antidepressant medications, n (%)					
Yes	NA	3 (5.3%)	13 (38.2%)	10 (55.6%)	0 (0%)
No	NA	16 (84.2%)	21 (61.8%)	8 (44.4%)	7 (100%)
Current medications, n					
Fluoxetine	NA	1	3	2	0
Sertraline	NA	1	2	2	0
Amitriptyline	NA	1	0	0	0
Desvenlafaxine	NA	0	2	2	0
Lithium carbonate	NA	0	1	1	0
Milnacipran	NA	0	1	1	0
Mirtazapine	NA	0	1	1	0
Escitalopram	NA	0	2	2	0
Duloxetine	NA	0	1	0	0
Escitalopram	NA	0	2	0	0
Venlafaxine	NA	0	1	0	0
Participants with comorbidities, n (%)	NA	11 (57.9%)	23 (67.6%)	11 (61.1%)	7(100%)
Comorbidities, n					
ADHD combined	NA	2	3	0	0
ADHD inattentive	NA	2	3	0	1
Migraine headaches	NA	1	0	0	0
Panic disorder	NA	1	3	2	1
Unspecified anxiety disorder	NA	0	3	1	1
Generalized anxiety disorder	NA	0	13	6	6
Social anxiety disorder	NA	0	10	4	6
Posttraumatic stress disorder	NA	1	0	0	0
Persistent depressive disorder	NA	2	2	0	0
Autism spectrum disorder	NA	0	1	1	0
Persistent motor tic disorder	NA	0	1	0	0
Cannabis use	NA	4	0	0	0
Alcohol use	NA	1	1 (in full sustained remission)	0	0

Abbreviations: ADHD, Attention Deficit Hyperactivity Disorder; ATHF, Antidepressant Treatment History Form; CDRS-R, Children’s Depression Rating Scale Revised; MDD, major depressive disorder; NA, not applicable; TMS, transcranial magnetic stimulation; TRD, treatment-resistant depression.

### Baseline MRI Comparison

**Figure 2.**
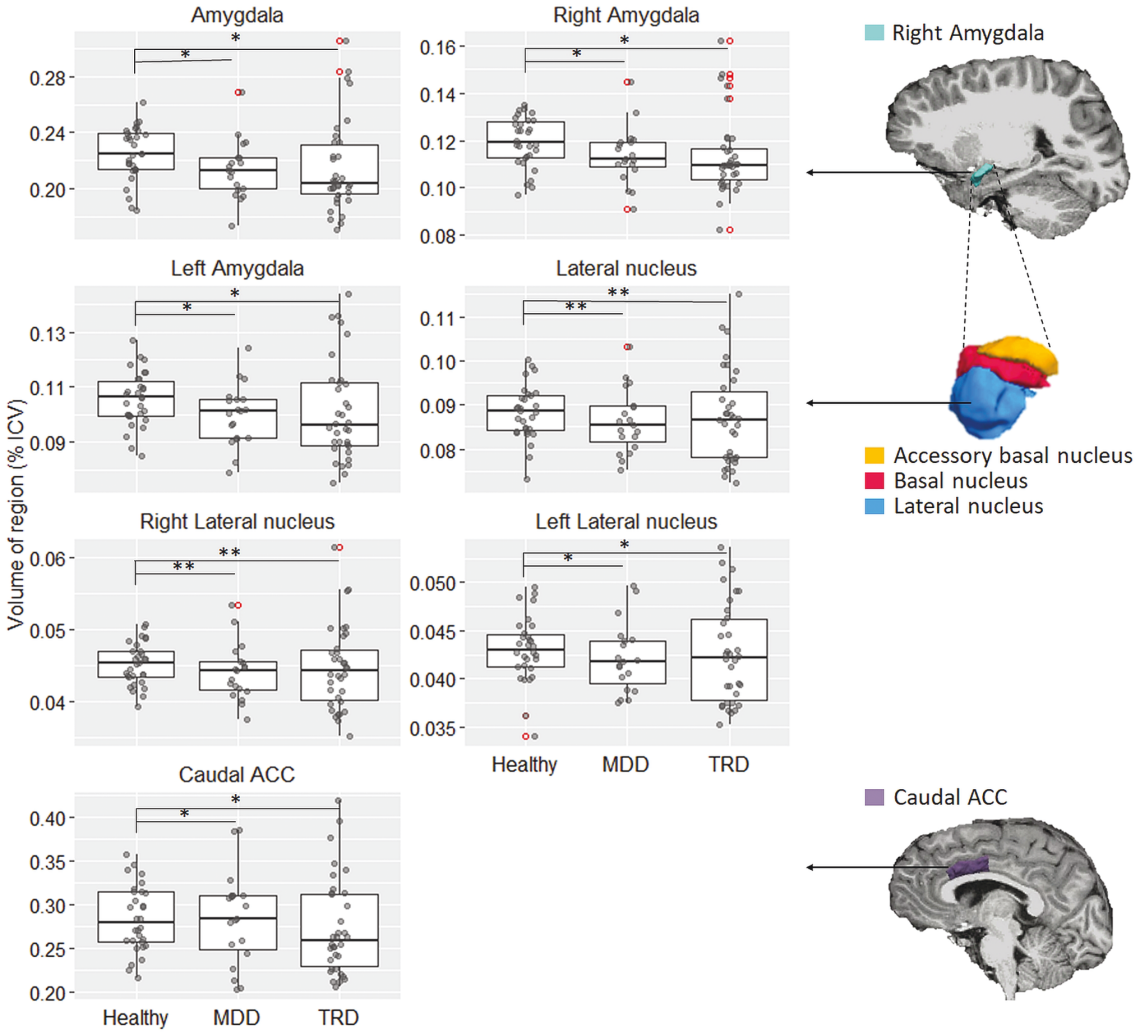
Subcortical and cortical volumes as a percentage to estimated intracranial volumes (% ICV) in healthy adolescents and adolescents with major depressive disorder (MDD) and treatment-resistant depression (TRD). In the box-and-whisker plots, the horizontal line inside the box represents the median volume (% ICV), the bottom and top edges reflect the interquartile range (25th and 75th percentiles, respectively), and the whiskers extend to the furthest datum within 1.5 times the interquartile range. False-discovery rate was used for multiple comparison correction. *P < .05; **P < .01.

There was a significant effect of depression (MDD and TRD) on whole amygdala volume after controlling for age, sex, and total baseline CDRS-R scores (ANOVA, *F*_[1,78]_ = 5.123, *P* = .026). Post hoc comparisons revealed that both MDD and TRD groups had significantly smaller amygdala volumes compared with healthy controls and that this difference was bilateral and related to the smaller volume of the lateral nucleus (Fig. 2; Table 2). Additionally, both patients with MDD and TRD had significantly smaller caudal anterior cingulate cortex volumes compared with healthy control. There were no differences in the volumes of the rostral anterior cingulate cortex and prefrontal regions between groups.

**Table 2.** Characteristics of patients with MDD and TRD and healthy comparison participants at baseline

Region	Baseline mean ± SD (percentage difference)	Method	Statistics
Amygdala	Healthy: .225 ± .019 MDD: .214 ± .021 (−5%) TRD: .215 ± .033 (−5%)	ANOVA	*F*_[1,78]_ = 5.123, *P* = .026
		Healthy vs MDD	t_77_ = −2.190, *P* = .032
		Healthy vs TRD	t_77_ = −2.261, *P* = .032
Right amygdala	Healthy: .119 ± .011 MDD: .114 ± .012 (−5%) TRD: .114 ± .017 (−5%)	ANOVA	*F*_[1,78]_ = 4.526, *P* = .037
		Healthy vs MDD	t_77_ = −2.039, *P* = .045
		Healthy vs TRD	t_77_ = −2.218, *P* = .045
Left amygdala	Healthy: .106 ± .010 MDD: .100 ± .011 (−5%) TRD: .101 ± .018 (−4%)	ANOVA	*F*_[1,78]_ = 4.348, *P* = .040
		Healthy vs MDD	t_77_ = −2.036, *P* = .050
		Healthy vs TRD	t_77_ = −1.992, *P* = .050
Lateral nucleus	Healthy: .0882 ± .0061 MDD: .0862 ± .0072 (−2%) TRD: .087 ± .011 (−1%)	ANOVA	*F*_[1,78]_ = 8.727, *P* = .004
		Healthy vs MDD	t_77_ = −2.882, *P* = .006
		Healthy vs TRD	t_77_ = −2.847, *P* = .006
Right lateral nucleus	Healthy: .0452 ± 0.0029 MDD: .0440 ± .0039 (−2%) TRD: .0446 ± .0058 (−1%)	ANOVA	*F*_[1,78]_ = 10.24, *P* = .0020
		Healthy vs MDD	t_77_ = −3.129, *P* = .003
		Healthy vs TRD	t_77_ = −3.048, *P* = .003
Left lateral nucleus	Healthy: .0431 ± .0034 MDD: .0421 ± .0036 (−2%) TRD: .0426 ± .0051 (−1%)	ANOVA	*F*_[1,78]_ = 6.190, *P* = .0150
		Healthy vs MDD	t_77_ = −2.418, *P* = .018
		Healthy vs TRD	t_77_ = −2.434, *P* = .018
Caudal anterior cingulate cortex	Healthy: .283 ± .038 MDD: .283 ± .053 (−.03%) TRD: .273 ± .055 (−3%)	ANOVA	*F*_[1,78]_ = 4.870, *P* = .0303
		Healthy vs MDD	t_77_ = −2.083, *P* = .041
		Healthy vs TRD	t_77_ = −2.488, *P* = .030

Abbreviations: MDD, major depressive disorder: TRD, treatment-resistant depression.

Mean ± SD are given as a percentage to estimated intracranial volumes. *P* values are corrected for multiple comparisons using the false-discovery rate method.

### Follow-Up Assessments

**Figure 3.**
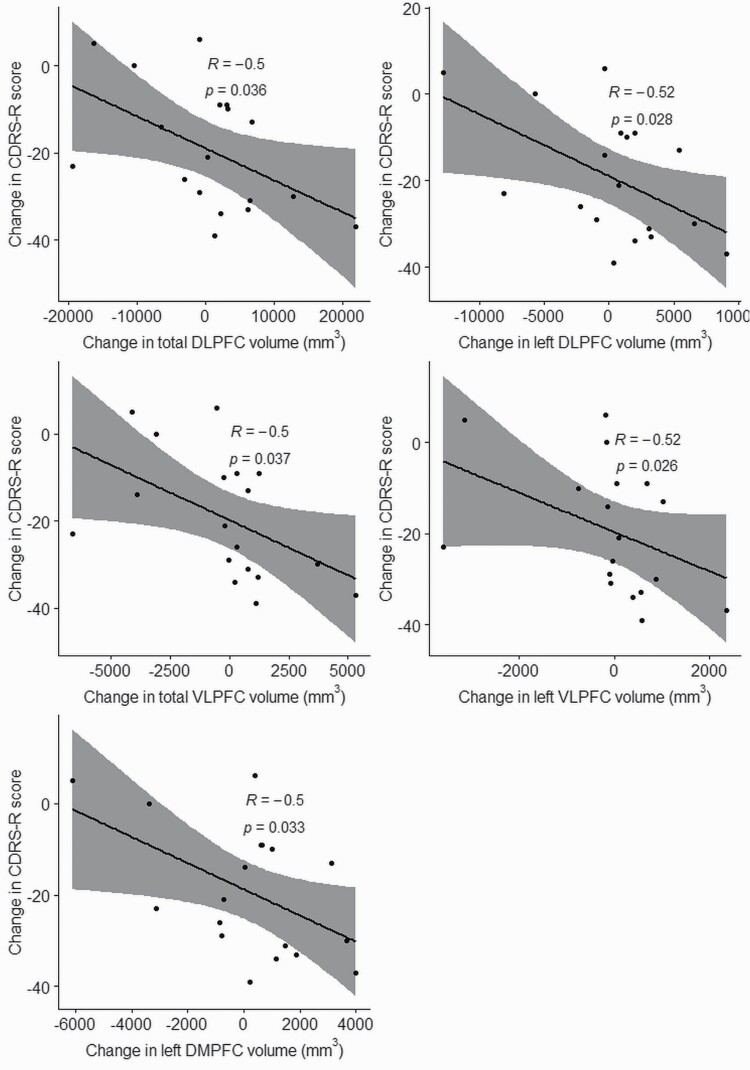
Correlations between change in volumes of prefrontal brain regions and change in total Children’s Depression Rating Scale, Revised (CDRS-R) scores of adolescents with treatment-resistant depression who received active transcranial magnetic stimulation treatment. Correlations for all regions were determined using Pearson correlation, except for the left VLPFC, which was determined using the Spearman’s rank correlation method. DLPFC, dorsolateral prefrontal cortex; DMPFC, dorsomedial prefrontal cortex; VLPFC, ventrolateral prefrontal cortex.

Paired tests between baseline and post active, left prefrontal, high-frequency TMS data showed a significant increase in the volume of whole amygdala, which was related to an increase in the right amygdala only (Table 3). No changes were detected in amygdala subfields. Active left prefrontal, high-frequency TMS also induced a significant increase in DLPFC volume, which specifically related to an increase in the left DLPFC only. Additionally, active TMS also induced a significant increase in the left DMPFC volume and a significant decrease in VLPFC volume, which specifically related to a decrease in the left VLPFC only. Decreases in CDRS-R scores were significantly correlated to increases in volume of DLPFC, left DLPFC, left DMPFC, VLPFC, and left VLPFC. There were no significant correlations between amygdala volume changes and decreases in CDRS-R scores (Fig. 3; Table 3). Additionally, an increase in the number of TMS sessions was not correlated with change in total CDRS-R scores (S = 1202, *P* = .336, R = −.241) but was significantly correlated with a decrease in Quick Inventory of Depressive Symptomatology Adolescent Self Report scores (S = 1435, *P* = .043, R = −.481). Sham left prefrontal, high-frequency TMS was not associated with any changes in cortical or subcortical volumes. The were no significant differences in percentage change in volumes different between active and sham groups.

**Table 3.** Subcortical and cortical volume changes associated with active TMS in adolescents with TRD

Region	Mean ± SD (mm^3^)	Statistics	Correlation
Amygdala	Baseline: 3219 ± 366 Post-TMS: 3349 ± 370 (+4%)	t_16.2_ = −4.038 *P* < .001	t_16_ = −.499 *P* = .624, R = −.124
Right amygdala	Baseline: 1702 ± 206 Post-TMS:1780 ± 213(+5%)	t_17.5_ = −3.739 *P* = .002	t_16_ = −.334 *P* = .743, R = −.083
DLPFC	Baseline: 105090 ± 17248 Post-TMS: 105601 ± 14713(+.5%)	t_17.8_ = −2.461 *P* = .024	t_16_ = −2.292 *P* = .036, R = −.497
Left DLPFC	Baseline: 53975 ± 8722 Post-TMS: 54205 ± 7274 (+.4%)	t_18.2_ = −2.425 *P* = .026	t_16_ = −2.414 *P* = .028, R = −0.517
VLPFC	Baseline: 25063 ± 3024 Post-TMS: 24866 ± 2830 (−0.8%)	t_20.1_ = −2.315 *P* = .031	t_16_ = −2.282 *P* = 0.037, R = −0.496
Left VLPFC	Baseline: 12439 ± 1472 Post-TMS: 12356 ± 1608 (−0.7%)	t_20.2_ = −2.118 *P* = .047	S = 1475.3 *P* = .026, R = −.522
Left DMPFC	Baseline: 27413 ± 4842 Post-TMS: 27589 ± 4376 (+.6%)	t_17_ = −2.237 *P* = .039	t_16_ = −2.339 *P* = .033, R = −.505

Abbreviations: DLPFC, dorsolateral prefrontal cortex; DMPFC, dorsomedial prefrontal cortex; TMS, transcranial magnetic stimulation; TRD, treatment-resistant depression; VLPFC, ventrolateral prefrontal cortex.

Paired *t* tests were corrected for the effect of age, gender and baseline CDRS-R scores. Spearman or Pearson correlations were performed (depending on the normality of the data) between change in volumes of brain regions and change in total CDRS-R scores.

## Discussion

This study is the first, to our knowledge, to examine cortical and subcortical volumetric differences in adolescents with TRD and MDD and healthy adolescents. This study also examined putative effects of left prefrontal, high-frequency TMS treatment in adolescents. Adolescents with TRD and current MDD had decreased amygdala and caudal ACC volumes compared with healthy adolescents. Further, left prefrontal, high-frequency TMS, but not sham, normalized the decreased amygdala volumes in adolescents with TRD and induced small increases in the volume of the stimulated left DLPFC. Taken together, these findings replicate previous reports of reduced amygdala volumes in depression (Nolan et al., 2020) and demonstrate that TMS may have frontolimbic neurostructural effects in adolescents with TRD. However, it is important to highlight the preliminary nature and inconsistencies of the findings. Adolescents with TRD and MDD had decreased amygdala volumes, and there were no correlations with amygdala volume changes and decreases in depressive symptoms in adolescents who underwent TMS. Conversely, adolescents with TRD and MDD did not have baseline differences in the DLPFC, but there was an increase in left DLPFC volume that correlated with a decrease in depressive symptoms in adolescents who underwent treatment. Although these findings are encouraging, large, prospective studies will be needed for definitive results.

Decreased cortical and subcortical volumes have been observed in adults with MDD and TRD (Klok et al., 2019). The anterior cingulate cortex (ACC) is implicated in salience assessment of emotional or motivational information while the amygdala plays a crucial role in emotional processing and vulnerability to depression (Stevens et al., 2011). Smaller ACC volumes have been consistently reported in patients with MDD and TRD (Bora et al., 2012; Klok et al., 2019), including in treatment-naïve adolescents with depression (Pannekoek et al., 2014) and TMS increases ACC volume in adults with TRD (Lan et al., 2016). Prior studies suggest that adolescents with MDD have reduced ACC volumes compared with healthy adolescent and adolescents with bipolar disorder (MacMaster et al., 2014). Other work demonstrated that adolescents with historical suicide attempts and non-suicidal self-injury have decreased ACC volumes (Ando et al., 2018). The present study is the first to our knowledge to specifically examine the ACC in adolescents with TRD. Our results suggest that an ACC volume deficit does not specifically characterize TRD in adolescents and further suggests that left prefrontal, high-frequency TMS may not restore ACC volume in adolescents. These findings are somewhat inconsistent with prior studies of adolescents with respect to disease burden (Ando et al., 2018) and conceptual models of TRD, antidepressant treatment response, and the ACC (Pizzagalli and Roberts, 2022).

Prior studies of amygdala volume changes in depression are variable, with studies reporting decreased, enlarged, or no difference in amygdala volumes (Hamilton et al., 2008; Nolan et al., 2020). For instance, the volumes of the amygdala and its nuclei were decreased in unmedicated (Tang et al., 2007) and recurrent patients (Sheline et al., 1998), whereas they were enlarged adults experiencing their first episode of MDD (van Eijndhoven et al., 2009) and in first-degree relatives of patients with MDD (Romanczuk-Seiferth et al., 2014). Nevertheless, the majority of MDD studies have reported smaller amygdala volumes in patients with MDD compared with healthy controls, showing approximately 5%−7% decreases in the left and right amygdala, respectively (Nolan et al., 2020). These findings are in line with the present study showing a bilateral decrease in amygdala volumes in depression, which is more pronounced in the right hemisphere. Additionally, the lateral nucleus of the basolateral amygdala complex may be particularly sensitive to chronic stress and early adversity (Zhang and Rosenkranz, 2016), which may explain the significant volume reduction in the lateral nucleus in MDD and TRD in the present study.

Interestingly, the smaller amygdala volumes in individuals with TRD compared with healthy controls was reversed by left prefrontal, high-frequency TMS, and this increase was associated with an increase in the volume of the stimulated left DLPFC. In accordance with our findings, previous studies have reported a significant increase in the volume of the amygdala (Zhou et al., 2020) and the left DLPFC (Smith et al., 2013) with antidepressant medications. Additionally, reduced DLPFC cortex volumes have been reported in adolescents with depression (Shad et al., 2012; Wehry et al., 2015). Although previous TMS studies have reported no or only near-significant increases in amygdala volumes in adults with MDD (Furtado et al., 2013; Hayasaka et al., 2017) and TRD (Dalhuisen et al., 2021), smaller pretreatment right amygdala volume has been associated with greater improvement in depressive symptoms with TMS treatment (Furtado et al., 2013). Given the cost and time associated with delivering TMS treatment, baseline predictive biomarkers such as the right amygdala volume deficits identified in the present study may identify patients who are most likely to benefit from the treatment.

Although this study provides new insight into cortical and subcortical volumetric changes in adolescents with TRD and the effect of TMS treatment, some limitations in our study warrant additional discussion. First, because this study combined MRI data from 4 different studies, several different MRI machines were used to acquire data and image acquisition differed across sites; however, this is not uncommon in studies of structural data in patients with MDD and obsessive-compulsive disorders (Schmaal et al., 2016; Boedhoe et al., 2017). Importantly, within-subject neurostructural data involved patients being scanned on the same scanner using the same protocol at baseline and posttreatment, so all within-participant comparisons eliminated the MRI scanner and protocol as a confounding variable. All studies used a standard vendor-product MPRAGE sequence with standard parameters, and within each study, all participants were scanned using the same scan protocol. Visual inspection of the FreeSurfer segmentations showed excellent parcellation of the cortex. Although everything possible was done to minimize the effects of scanner heterogeneity in the dataset, this remains a limitation of the study. Second, the sample size of the MDD, active TMS, and sham TMS groups were small. Therefore, the baseline structural differences and changes with TMS should be interpreted with caution. Third, the stability of TMS-induced volumetric changes cannot be determined due to the absence of long-term follow-up timepoints. The acute volumetric changes may represent transient, persistent, or progressively increasing treatment-related changes. Fourth, prior antidepressant use is a confounding factor because it has been suggested to alter cortical and subcortical brain volumes (Fossati et al., 2004; Dusi et al., 2015). Additionally, although greater volumetric gains may be seen in medication-free and non-treatment resistant adults with depression, TMS is frequently used in TRD, and therefore it remains an ethical, clinical, and scientific priority to explore its effects in adolescents with TRD. Fifth, the definition of TRD in the present study may not have provided the opportunity to examine a highly treatment refractory adolescent sample and this may explain the limited volumetric differences among patients with TRD and MDD in the current study. Sixth, navigation methods varied among the depressed adolescents in the study treated with TMS, and this may have been a confounding factor. Finally, because this study examined pooled data from several studies, the characterization of TRD and MDD across studies had differences that could have impacted the present findings.

Despite these limitations, we found evidence suggesting that high-frequency left prefrontal TMS in adolescents with TRD produces volumetric changes under the coil and in other regions that subserve mood regulation. Bilateral amygdala volumes were reduced in adolescents with MDD and TRD by approximately 5% compared with healthy adolescents, and the right amygdala volume increased by 5% in adolescents with TRD after treatment. Based on our results, structural changes in adolescents with depression and the effects of left prefrontal, high-frequency TMS in adolescents with TRD may be similar to those observed in adults with depression. Future efforts should focus on developing biomarkers to differentiate MDD from TRD in adolescents and guide treatment with TMS. Resting-state connectivity biomarkers may be a promising approach that could prove scalable for clinical practice (Cullen et al., 2014).

## Interest Statement

Dr Demitrack is a consultant to Neuronetics and a full-time employee of Trevena, Inc. Ms. Heart is employed by Advicenne, Inc. She is a former employee of Neuronetics with minor stock investment. Dr Strawn has received research support from AbbVie, Neuronetics, Otsuka Pharmaceutical Co, Ltd, National Institute of Mental Health, National Institute of Child Health and Human Development, National Institute of Environmental Health Sciences, and the Yung Family Foundation. He receives royalties from Springer Publishing Co and has received honoraria from CMEology, Neuroscience Education Institute, and Genomind. He receives royalties from UpToDate. He has received material support from and provided consultation to Myriad Genetics. Dr Port serves as an imaging consultant for Takeda Pharmaceutical Company, Ltd. and Bioclinica. Dr Croarkin has received research support from the National Institute of Mental Health, Pfizer, Neuronetics, Inc. and NeoSync, Inc. He has received equipment support from Neuronetics, Inc. and MagVenture, Inc. for investigator-initiated research. He has received material support from and provided consultation to Myriad Genetics. He has consulted for Engrail Therapeutics, Myriad Genetics, Procter & Gamble Company, and Sunovion. The other authors declare no competing interests.
